# P02-029 - CAPS or SJIA

**DOI:** 10.1186/1546-0096-11-S1-A136

**Published:** 2013-11-08

**Authors:** E Alekseeva, R Denisova, S Valieva, T Bzarova, T Sleptsova, E Mitenko, K Isayeva

**Affiliations:** 1 Rheumatology, Scientific Center Of Children’s Health of RAMS, Moscow, Russian Federation

## Introduction

Systemic Juvenile Idiopathic Arthritis (sJIA) is chronic disease. Some patients are resistant to standard immunosupressivetherapy and anti IL6 treatment. Some of these patients have autoinflammatory disease.

## Case report

The patient became ill when she was 3 years old. She had fever, rash and artralgia and pericarditis. After examination in hospital she was diagnosed sJIA. She took glucocorticosteroids 1 mg/kg daily per os, methylprednisolone 10 mg/kg IV, №3, and MTX 10mg/m2/week with positive results. She took that therapy during 2 years. When the dose of GC decreased she had flares (fever, rash, arthritis). In 2 years after beginning she was examined for TRAPS. She took infliximab without improving. The analysis for TRAPS was negative. When she took GC 0,5 mg/kg/day she had sever flare and tocilizumab treatment was initiated. The fever disappeared and CRP was normal but rash was persisted. She took tocilizumab 10 months and dose of GC was decreased to 0,05 mg/kg/day. After 10 months of tocilizumab treatment she had toxic allergic reaction and tocilizumab was canceled. She was examined for CAPS and mutation in gene **NLRP3** - c.2113C>A in geterozygota. In that time there was no anti IL-1 medicines in Russia so she was given cyclosporine and MTX was continued. When she was 7 years old for flare canacinumab therapy was initiated in dose 4 mg/kg. The fever, rash, arthritis were disappeared and CRP and ESR became normal. After 3 months GC was cancelled. After 4 canacinumab injection she had flare and puls therapy of GC IV was performed with positive results.

## Discussion

So it is still question if she has CAPS or sJIA. Because She is not in remission on anti IL 1 therapy and also GC therapy is effective for this patient and on the other hand she has mutation in the cryopirin gene.

## Disclosure of interest

None declared.

